# Epstein-Barr Virus Positive B-Cell Lymphoproliferative Disorder of the Gastrointestinal Tract

**DOI:** 10.3390/cancers13153815

**Published:** 2021-07-29

**Authors:** Eri Ishikawa, Akira Satou, Masanao Nakamura, Shigeo Nakamura, Mitsuhiro Fujishiro

**Affiliations:** 1 Department of Gastroenterology and Hepatology, Nagoya University Graduate School of Medicine, Nagoya 466-8560, Japan; makamura@med.nagoya-u.ac.jp (M.N.); mtfujish@med.nagoya-u.ac.jp (M.F.); 2 Department of Surgical Pathology, Aichi Medical University Hospital, Nagakute 480-1195, Japan; kei_akira0121@hotmail.com; 3 Department of Pathology and Laboratory Medicine, Nagoya University Hospital, Nagoya 466-8550, Japan; snakamur@med.nagoya-u.ac.jp

**Keywords:** Epstein-Barr virus (EBV), mucocutaneous ulcer, diffuse large B cell lymphoma (DLBCL), lymphoproliferative, gastrointestinal lymphoma, gastric lymphoma, intestinal lymphoma, programmed cell death ligand 1 (PD-L1)

## Abstract

**Simple Summary:**

Epstein-Barr virus (EBV) contributes to the reactive and neoplastic lymphoid proliferation of B-, T-, and NK-cell lineages, which represent a vast clinicopathological spectrum ranging from indolent, self-limited disease to aggressive lymphomas. EBV-positive B-cell lymphoproliferative disorder (EBV^+^ B-LPD) is the most common of these diseases, accounting for 3% to 15% of diffuse large B-cell lymphomas. The spectrum of EBV^+^ B-LPD is expanding with advances in understanding immunosenescence and iatrogenic immunodeficiency in the era of immune-oncology. We review EBV^+^ B-LPD affecting the gastrointestinal tract with a focus on the PD-L1 expression in tumor and non-malignant immune cells to better understand this peculiar disease.

**Abstract:**

Epstein-Barr virus positive B-cell lymphoproliferative disorder (EBV^+^ B-LPD) encompasses a broad clinicopathological spectrum and distinct clinical behavior that relatively favors the gastrointestinal (GI) tract. In this review, we provide an update on the clinicopathological features and biological behavior of EBV-positive mucocutaneous ulcer (EBVMCU) and primary EBV^+^ diffuse large B-cell lymphoma (DLBCL) of the GI tract. EBVMCU is a newly recognized entity but well known as an indolent and self-limited EBV^+^ B-LPD occurring in various immunodeficiencies. In contrast, EBV^+^ DLBCL constitutes the largest group of EBV^+^ B-LPDs and is regarded as an aggressive neoplasm. These two distinct diseases have historically been distinguished in the reappraisal of age-related EBV-associated B-LPDs but are challenging in routine practice regarding their differential diagnostic and therapeutic approaches. An increasing number of reports indicate that they are epidemiologically prevalent beyond western and eastern countries, but their comprehensive analysis is still limited. We also describe the PD-L1 positivity of tumorous large cells and non-malignant immune cells, which is relevant for the prognostic delineation among patients with primary DLBCL of the GI tract with and without EBV on tumor cells.

## 1. Introduction

Epstein-Barr virus (EBV) is a gamma herpes virus that infects the majority of the world population. EBV induces B-cell transformation, and disruption of a finely balanced relationship between the virus and host immune system can lead to EBV^+^ B-cell lymphoproliferative disorders (B-LPDs), which represent a wide and expanding clinicopathological spectrum ranging from indolent and self-limited disease to aggressive lymphoma.

Dojcinov et al. recently divided EBV^+^ B-LPDs into five categories: infectious mononucleosis (IM); EBV^+^ DLBCL, not otherwise specified (NOS); EBVMCU; DLBCL-associated chronic inflammation (DLBCL-CI); lymphomatoid granulomatosis (LyG) [1]. IM is an acute clinical manifestation of EBV infection characterized by reactive and self-limiting lymphoproliferation in a minority of immunocompetent adolescents [2], whereas chronic active EBV infection may have non-lymphomatous lymphoproliferative lesions in the gastrointestinal (GI) tract [3,4,5].

EBV^+^ DLBCL was initially described as senile EBV-associated B-LPD by Oyama et al. in 2003 [6] and was listed as DLBCL of the elderly in the 2008 WHO classification [7,8,9]. After the original study, the development of nodal EBV^+^ DLBCL in young patients with no evidence of immunosuppression was reported by Nicolae et al. in 2015 [10]. As a result, the 2017 WHO classification of malignant lymphoma encompassed these diverse diseases and emphasized that EBV^+^ DLBCL, NOS often affects both young and elderly immunocompetent patients. EBV^+^ DLBCL often presents an aggressive clinical course with frequent extranodal disease. Primary EBV^+^ DLBCL of the GI tract (giDLBCL) accounted for 5% to 10% of consecutively diagnosed giDLBCL in a series of 62, 107, and 240 cases [11,12,13]. Although contradictory conclusions on the significance of EBV in regards to clinical outcome have been reported [10,14,15,16,17], we recently documented the negative impact of EBV in the largest series of 156 patients with gDLBCL and 51 patients with iDLBCL in the rituximab era to date [11,12].

EBVMCU is an ulcerating EBV^+^ B-LPD with a self-limited indolent course [18]. The disease is associated with advanced age and immunosuppression, such as primary immunodeficiency, post-transplantation, and other iatrogenic causes, including methotrexate (MTX), prednisolone (PSL), azathioprine (AZA), cyclosporin A (CYA), and TNF-α antagonists [18]. The disease often involves the oropharyngeal mucosa, skin, and GI tract. In particular, EBVMCU in the GI tract (giEBVMCU) is frequently detected in patients with inflammatory bowel disease or immune-related colitis, which is the specific disease in the intestine [18,19,20,21,22,23,24]. In general, giEBVMCU responds well to conservative management, but patients with immune-related colitis are distinct from others in the high frequency of perforation requiring surgery [24].

DLBCL-CI is a rare EBV-associated entity described in association with pyothorax with a history of underlying chronic inflammation and pursues an aggressive clinical course [25]. Similarly, LyG is a rare angiocentric and angiodestructive EBV^+^ B-LPD associated with at least some degree of inherent immunosuppression. LyG frequently occurs in lung and skin/subcutaneous tissue. GI tract involvement is rare in patients with DLBCL-CI or LyG [26,27]. Therefore, they are beyond the scope of the present review.

Recent advances in immune-oncology have expanded our knowledge of immune evasion, mostly by programmed cell death ligand 1 (PD-L1) and the PD-1 pathway has become an attractive therapeutic target in various malignancies [28,29,30,31,32]. We also showed that PD-L1 immunohistochemistry aids in the differential diagnostic approach for EBV^+^ B-LPDs and their morphological analogues [33]. EBV^+^ DLBCL is accompanied by high PD-L1 expression but the clinicopathological significance remains to be clarified [29,34]. We further revealed that PD-L1 expression on tumor cells and non-malignant immune cells has an opposite prognostic impact in patients with giDLBCL [12,35].

The focus of this review is to describe the clinicopathological features of EBV-associated lymphoproliferative disorders (LPDs), especially EBVMCU and EBV^+^ DLBCL, in light of the GI tract involvement and PD-L1 expression.

## 2. EBV Biology and the GI Tract

EBV was the first oncogenic virus identified and it persistently infects the B cells of >95% of adults, resulting in an asymptomatic life-long carrier status [36]. The EBV life cycle is biphasic, with phases of lytic replication and latency [37]. EBV is etiologically linked to a wide range of human tumors, including gastric carcinoma, nasopharyngeal carcinoma, and lymphoma, and three EBV latency patterns have been recognized [37,38,39]. Latency I is associated with Burkitt’s lymphoma and a distinct subset of gastric carcinomas, whereas Latency II is associated with classic Hodgkin’s lymphoma, extranodal NK/T-cell lymphoma, and nasopharyngeal carcinoma [40]. Latency III is linked to immunodeficiency-associated LPDs arising in the setting of immunosuppression due to HIV infection, post-transplantation, and other iatrogenic causes, such as MTX and anti-TNF-α therapy.

Some studies have reported that the presence of EBV is restricted to lymphoid cells, not benign epithelial cells, in gastritis lesions and normal colonic mucosa [41,42]. In the stomach, a positive correlation between EBVDNA load and *Helicobacter pylori* positivity has been reported, which suggests that the *H*. *pylori* infection could trigger EBV to switch from the latent to lytic phase of its life cycle [43,44]. In addition, EBV infection of tumor cells is detected in approximately 9% of gastric cancer (GC) cases; it is categorized as one of the major GC types and characterized by a high frequency of amplification and elevated expression of PD-L1 [45,46,47]. Notably, EBV^+^ GC is an attractive target for anti-PD-1/PD-L1 therapy in the current era [48,49].

In the intestine, EBV replication has been reported to be associated with severe inflammatory bowel disease and mucosal inflammation [50]. The presence of EBV-infected B lymphocytes in colonic lesions of IBD may indicate a potential role of EBV in colonic immune disturbances, which may be caused by the inflammatory process itself, immunosuppressive medication, or a combination of both. In contrast, Lopes et al. showed that mucosal EBV load does not correlate with the presence of inflammation or endoscopic severity despite a higher prevalence of EBV infection in IBD [51]. Whether EBV is involved in the pathogenesis or is an innocent bystander remains unclear, but active inflammation with intramucosal expansion of EBV-infected B-lymphocytes in IBD patients may cause local impairment. In IBD patients exposed to thiopurines or anti-TNF agents, particularly in combination, the risk of LPDs, mostly associated with EBV, is higher [52,53,54]. Thiopurines are cytotoxic for NK and cytotoxic T cells, which restrict proliferation of EBV-infected and immortalized B cells, which could be associated with lymphomagenesis [55].

## 3. EBV-Positive Mucocutaneous Ulcer (EBVMCU)

EBVMCU, first recognized by Dojcinov et al., is defined as an ulcerating EBV^+^ B-LPD affecting the skin and mucosal surfaces, with a typically indolent course and spontaneous regression in some cases.

### 3.1. Clinical Features

EBVMCU occurs in iatrogenic immunosuppressed patients with autoimmune disorders and inflammatory bowel disease receiving MTX, CYA, AZA, TNF-α antibody, tacrolimus (Tac), or steroid treatment, in solid organ or bone marrow transplant recipients, in HIV-positive patients, after other lymphoma or tumor treatment, and in elderly patients. Elderly subjects are markedly restricted and deficient in their epitope-specific T-cell repertoire, leading to an increased risk of infection for the host [18,56].

The disease manifests as shallow, sharply circumscribed ulcers located mostly in the oropharyngeal mucosa, skin, and GI tract without associated systemic symptoms, lymphadenopathy, or involvement of any other site. A large superficial non-healing wound is an important diagnostic clue, and biopsy is warranted to determine the underlying pathology. Note that lesion may be underestimated as a non-specific ulcer in routine endoscopic diagnosis or over-diagnosed as monomorphic large B-cell proliferation (i.e., DLBCL) and simply polymorphic PTLD in pathological diagnosis, without recognition of iatrogenic induced EBVMCU. In a Japanese cohort of 34 patients with EBVMCU, 90% of the cases received immunosuppressants, such as MTX or hydroxycarcamide [57]. The diseases were frequently identified in the gingiva or tonsil, and a small number of cases had multiple ulcers.

In the setting of solid organ transplant-related immunosuppression, Hart et al. identified EBVMCU in 10% of transplant recipients with EBV^+^ post-transplant lymphoproliferative disorder (PTLD) [58]. In their cohort, all seven patients with EBVMCU received solid organ transplants, but not hematopoietic stem cell transplants. EBVMCU may not develop in patients who receive a great level of immunosuppression, which prevents a localized response to EBV infection. Interestingly, they reported that none of the patients had EBV DNA detectable in the blood, and the patients remained PCR-negative in the blood during the follow-up period despite the biopsies being strongly positive by EBER-ISH. The lack of quantifiable EBV viremia may be an additional distinguishing feature of EBVMCU but should be validated in a future series.

### 3.2. Pathological Features

The lesions exhibit polymorphous infiltration of plasma cells, lymphocytes, histiocytes, and eosinophils, with atypical large B-cell blasts with Hodgkin/Reed-Sternberg (HRS) cell-like morphology [18]. Focal necrosis and angioinvasion are frequently seen. The large cell-predominant pattern may mimic DLBCL in a subset of cases. These large transformed cells are partially positive for CD20 and exhibit PAX5, Oct-2, MUM1, CD30, and EBER positivity, but are CD10 and BCL6 negative with a non-germinal center immunophenotype. CD15 is positive in 50% of cases. The background T cells consist of CD4-positive and CD8-positive lymphocytes. Less than 50% of EBVMCU cases have clonal Ig gene rearrangements or clonal T-cell rearrangement.

### 3.3. PD-L1 Expression

A handful of studies have reported PD-L1 expression in EBVMCU. Satou et al. described the lack of PD-L1 expression on tumor cells in seven cases with MTX-associated EBVMCU using two different clones (SP142 and E1J2J) [59], which is consistent with findings reported by Daroontum et al. in 13 cases of EBVMCU associated with treated lymphoma or MTX [60]. Interestingly, in their subsequent series, one possibly exceptional EBVMCU case in which multiple EBV-driven B-LPDs developed after spontaneous regression of the disease was found to have PD-L1 expression on tumor cells at the time of the initial onset of EBVMCU [61]. This PD-L1 expression detected by immunohistochemistry (clone SP142) may be related to unusual clinical behavior after the spontaneous regression of EBVMCU. In contrast, Prieto et al. reported that all three cases with EBVMCU were positive for PD-L1 on large cells and HRS-like cells when using clone 28-8 [23]. This discordance in PD-L1 expression between studies is thought to be due to the use of different anti-PD-L1 antibody clones (SP142 vs. 28-8). However, a definitive conclusion cannot be drawn because of a limited number of cases examined, and this issue is expected to be clarified in a larger study.

### 3.4. Treatment and Clinical Course

Repeatedly, EBVMCU is characterized by a self-limited indolent course; generally, spontaneous remission after reduction or discontinuation of immunosuppression as the initial treatment within approximately 8 weeks. This is distinct from the behavior in EBV^+^ DLBCL, though some patients with EBVMCU occasionally require therapeutic intervention, including standard chemotherapy and radiotherapy [18,23,57,59,62]. Therefore, it is essential for clinicians to consider EBVMCU in the differential diagnosis of mucocutaneous ulcers to avoid excessive treatment. Although the reduction of immune suppression is not possible in age-related cases without any additional iatrogenic immunosuppression, approximately 60% are expected to have spontaneous complete remission during the clinical course [18]. Interestingly, patients with treated lymphoma-associated EBVMCU have been reported to have worse survival than patients with MTX-associated EBVMCU because of lymphoma relapse or unrelated causes [60]. EBVMCU in the setting of treated lymphoma may be predictive of an aggressive clinical course.

### 3.5. giEBVMCU

In the English language literature, 30 cases of giEBVMCU have been reported (Table 1) [18,19,20,21,22,23,24,58,60,63,64,65,66,67,68,69,70,71,72]. The most common clinical setting is iatrogenic immunosuppression in the context of inflammatory bowel disease, followed by post-organ transplant, immune-related colitis (irColitis), rheumatoid arthritis (RA), and treated-lymphoma (Table 2). The other patients had autoimmune thrombocytopenia, HIV, post-hematopoietic stem cell transplantation, hypogammaglobulinemia, and rheumatic polymyalgia or were associated with advanced age. Twenty (67%) of the 30 patients were receiving therapeutic immune suppression, including AZA, infliximab (IFX), CYA, MTX, mycophenolate mofetil (MMF), PSL, and Tac. The colon was the most common site (Table 2) The significance of the difference between single and multiple lesions is currently unclear.

#### 3.5.1. giEBVMCU in Patients with IBD

Six patients with EBVMCU in IBD consisted of three with Crohn’s disease, two with ulcerative colitis, and one without any available information. Except for the one with Crohn’s disease presenting with an anal lesion, all cases had rectal involvement, two of which were accompanied by synchronous involvement of the colon. Macroscopically, most cases had ulcerated lesions during treatment with immunosuppressive regimens, including AZA, IFX, MTX, 6-mercaptopurine, and CYA, whereas only one used mesalazine alone and had a non-specific erythematous lesion. Interestingly, two cases had multiple lesions, which is not typical for ordinal EBVMCU [20,22]. In most EBVMCU cases with IBD, the clinical course was benign with complete recovery by reduction of immune suppression or aggressive therapy. Two patients achieved complete remission upon reduction of immune suppression. One patient with ulcerative colitis received rituximab because colonic biopsies showed persistent necrotic ulceration with EBV-positive immunoblasts after 4 weeks, though the ulcer slightly decreased in size by reduction of immune suppression and achieved complete remission [22]. The other patient with Crohn’s disease finally underwent proctectomy with terminal colostomy due to an inability to control the rectal symptoms despite cessation of AZA and IFX [21]. The third patient using only mesalazine did not receive any therapy and presented with stable persistent disease without symptomatology for 6 months [23]. The remaining patient with Crohn’s disease treated with AZA and adalimumab in the past presented an aggressive clinical course, developing widespread classic Hodgkin lymphoma 18 months after cessation of IFX and MTX [20].

Although the treatment strategy for IBD after regression of LPD is not well established, reintroduction of immune suppression is usually avoided to prevent relapse of the LPD. However, long-term follow-up data on disease activity in IBD are still not available among patients with giEBVMCU, and Satou et al. recently indicated that most RA patients suffer from exacerbation of the disease with polyarticular inflammation after 2 months after withdrawal of immune suppression [59]. Guidelines or recommendations for treatment after the regression of a LPD should be developed in IBD patients with EBVMCU.

#### 3.5.2. giEBVMCU in Patients with Organ Transplant

Hart et al. and Isnard et al. reported on five solid organ transplant recipients with giEBVMCU [58,69]. Four patients were immunosuppressed after kidney transplants and the remaining one after a lung transplant. Three of these patients had EBVMCU in the colorectum and one each in the esophagus and terminal ileum. The duration of immunosuppressive therapy before symptoms onset ranged from 8 to 72 months (median 24 months). All of the patients achieved complete remission with reduction of immune suppression and additional rituximab therapy.

#### 3.5.3. giEBVMCU in Patients with irColitis

Pugh et al. reported that four (25%) of 16 patients with irColitis had EBVMCU of the colorectum. All of the cases presented with multiple well-defined, punched-out, crateriform ulcerating lesions with normal intervening mucosa and perforation. As perforation rarely occurs in patients with EBVMCU in other settings, this punched-out ulcerating lesion leading to perforation may be a clinical hallmark of EBVMCU in irColitis patients [24]. All four cases received anti-CTLA-4 treatment for melanoma, and one of them was treated with a combination of anti-CTLA-4 and anti-PD1 regimens. One patient had steroid-refractory colitis with additional IFX therapy. All had a favorable clinical course after surgical resection of the perforated giEBVMCU, except for one who experienced a deteriorated clinical course after the operation. In this cohort, EBV-positive lymphoid cells were identified at the ulcer base in surgically resected specimens. Therefore, we should pay attention to the possibility of sampling error in endoscopic biopsy specimens of EBVMCU. Interestingly, one case had a Crohn’s-like cobblestone appearance with multiple fissuring ulcerations in the small intestine. The authors indicated that the diversification of the T-cell repertoire, brought by CTLA-4 blockade, is associated with immune-related adverse events. They also described that the diversification inhibits EBV-induced lymphoma growth in both mice and humans [73,74,75], which appears to be contradictive with the development of EBVMCU in the setting of irColitis. The giEBVMCU arising in patients with irColitis may not be caused by immunodeficiency, and mechanism of their development should be clarified in the future. Patients with this irColitis-related giEBVMCU are expected to increase in number, and further studies of this peculiar disease are needed.

#### 3.5.4. giEBVMCU in the Other Patients

Dojcinov et al. and Nomura et al. reported on three patients with RA and giEBVMCU [18,68]. The immune suppression regimens included MTX, AZA and PSL. One of the cases evaluated had esophageal ulcer with complete remission after reduction of immune suppression. Another case had a jejunal ulcer and good clinical course but underwent surgical resection because of perforation.

**Figure 1:**
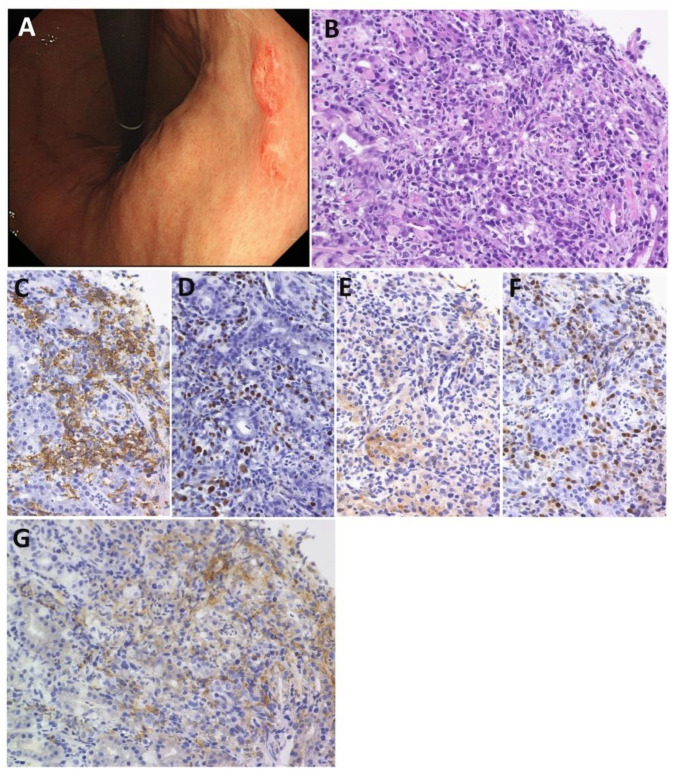
Histological features of EBV-positive mucocutaneous ulcer (EBVMCU). (**A**) Endoscopic image revealing small shallow ulcers in the stomach. (**B**) The infiltrate is polymorphous, containing lymphocytes, histiocytes, and immunoblasts, but not Hodgkin-like cells. (**C**) The scattered large cells were positive for CD20, (**D**) EBER, (**E**) CD30, and (**F**) MUM1. (**G**) PD-L1 is expressed on nonmalignant immune cells, but not on tumor cells. Original magnifications: ×200 (**B**–**G**).

Daroontum et al. reported two cases with treated lymphoma-associated giEBVMCU in the stomach (Figure 1) and intestine within 1 year of the cessation of chemotherapy for adult T-cell leukemia/lymphoma. They prompted us to be aware of EBVMCU in the setting of intensively treated hematolymphoid malignancy [60]. Both of our cases achieved complete remission on the reduction of immune suppression or without any therapy, but one of them died due to complications 4 months after allogeneic transplantation. Karube et al. also reported a patient with treated lymphoma-associated giEBVMCU with a history of classic Hodgkin lymphoma, who was unique in having multiple ulcerative and unusually elevated lesions in the large intestine, followed by EBV^+^ DLBCL in the liver [67]. PCR analysis of the immunoglobulin heavy chain gene rearrangement revealed that all lesions were clonally distinct, suggesting that histologically and clonally distinct B cells can simultaneously proliferate in the EBV-associated setting.

Zanelli et al. reported on two immunocompetent patients with giEBVMCU in advanced age diagnosed based on the surgically resected specimen for colonic diverticulitis. One of these patients was treated with immunosuppressive agents, including MTX and PSL [65,72]. In addition to advanced age, chronic and localized mucosal irritation from diverticulitis may favor the localized proliferation of EBV-infected cells.

## 4. EBV-Positive Diffuse Large B-Cell Lymphoma (EBV^+^ DLBCL)

EBV^+^ DLBCL, NOS was first described in Japan, and is defined as an EBV-positive clonal B-cell lymphoid proliferation in the 2017 WHO classification. Other well-defined lymphoma entities, such as lymphomatoid granulomatosis, acute or recent EBV infection, plasmablastic lymphoma and DLBCL associated with chronic inflammation, and EBVMCU, are excluded from this category.

### 4.1. Clinical Features

EBV^+^ DLBCL was originally documented among elderly patients >60 years of age [6,7]. Most patients present with extranodal, clinically aggressive disease and have no history of immunosuppression. EBV has been reported in 3–15% of patients with DLBCL [7,8,9,76]. It is more prevalent in East Asia and relatively rare in Western countries. Although a lack of uniform criteria, including the percentage of EBV^+^ tumor cells required for diagnosis, accounts for reported differences in disease prevalence and prognostic features [14,15,16], a threshold of 80% for the diagnosis of EBV^+^ DLBCL is recommended in the 2017 WHO classification to avoid the inclusion of cases in which EBV may be a bystander in non-neoplastic cells.

Elderly patients with EBV^+^ DLBCL, NOS exhibit a high prevalence of B symptoms (35–50%) and extranodal disease (40–70%), including skin, lung, and GI tract [7,77,78]. The pathogenetic mechanism in elderly patients is thought to be immunosenescence, a complex spectrum of regressive changes that affect immune competence and immune surveillance over EBV infection as a result of age. T-cell responses appear to be the most profoundly affected. The naive CD8^+^ EBV-specific T-cell pool is diminished and replaced by senescent and functionally inferior effector memory cells, resulting in a reduction of the T-cell antigenic repertoire. Such changes generate an environment similar to iatrogenic immunosuppression [79,80,81]. In contrast, patients ≤45 years of age with EBV^+^ DLBCL, NOS have nodal disease, with only 11% exhibiting extranodal involvement [10].

The addition of rituximab to anthracycline-based chemotherapy has improved the survival outcome in patients with common DLBCL [82,83]. The treatment strategy for EBV^+^ DLBCL is usually in concordance with the current approach for common DLBCL. Elderly patients with EBV^+^ DLBCL, NOS have poor survival, which is clearly contrasted by a favorable outcome among young patients with a nodal disease [6,7,10,15]. Some studies have documented contradictory conclusions regarding with the prognostic impact of EBV among DLBCL patients in the rituximab era because of differences in age, the different percentages of EBV harbored on tumor cells for diagnosis, and the small number of enrolled cases [14,15,16,17,84].

### 4.2. PD-L1 Expression

Among patients with common DLBCL, the frequency of PD-L1 expression has been reported to be 6% to 26% with different cut-off values and anti-PD-L1 monoclonal antibodies [34,85,86,87]. In general, an alteration in chromosome 9p24.1 is rarely found, and the structural variations disrupting the 3′ untranslated region of the PD-L1 gene, which correlates with PD-L1 expression, is detected in 8% of common DLBCL cases [85,88]. However, whether this PD-L1 expression on malignant tumor cells has an adverse prognostic impact is still controversial [34,86,89]. PD-L1 is also expressed on nonmalignant immune cells, such as macrophages and dendritic cells in DLBCL; thus, the prognostic significance is unproven because of the paucity of reports.

In EBV^+^ cases, EBV-LMP1 increases PD-L1 promoter and enhancer activity [90]. EBV^+^ DLBCL has a higher frequency of PD-L1 expression on tumor cells (19–100%) and immune cells (40–100%) compared to EBV-negative DLBCL [29,34,89]. Notably, 76% of young patients with EBV^+^ DLBCL exhibit PD-L1 positivity in the tumor cells [10]. Takahara et al. recently reported that PD-L1 expression (clone SP142-positive staining) was present in more than 5% of tumor cells in only 6 (11%) of 57 cases (95% were >45 years old) [91], clearly contrasting the 77% reported in younger cases (<45 years old) [10]. The former also indicated that PD-L1^+^ cases had significantly shorter progression-free survival (*p* = 0.002) and relatively short overall survival (*p* = 0.26), compared to PD-L1-negative cases.

### 4.3. Overall Perspective of Primary giDLBCL as a Control Cohort

DLBCL is the most common lymphoma type affecting the GI tract, accounting for 39–58% of cases [92,93,94]. More than 70% of giDLBCL cases occur in the stomach [35,92]. In intestinal DLBCL (iDLBCL) cases, the most frequently involved site is the ileocecum, and approximately 10% have histological transformation of intestinal follicular lymphoma [12,13,95]. In gastric DLBCL (gDLBCL) cases, *H. pylori* negativity, advanced Lugano stage, elevated serum lactate dehydrogenase (LDH), multiple gastric lesions, B symptoms, and EBER positivity have been reported to be adverse prognostic factors, whereas IGH-involved translocations are favorable prognostic factors [11,96,97,98,99]. On the other hand, in iDLBCL cases, advanced age, perforation, microenvironment PD-L1 negativity, and EBER positivity have been shown to be adverse prognostic factors. Surgical resection followed by chemotherapy has been identified as a favorable prognostic factor among those patients [12,95,100]. Although the 5-year overall survival rates of gDLBCL and iDLBCL in the rituximab era are 88% and 71%, respectively, the anatomical site was not a prognostic factor in an entire series of giDLBCL [35].

### 4.4. Primary EBV^+^ giDLBCL

#### 4.4.1. Clinical Features of Primary EBV^+^ giDLBCL

Few studies have focused on EBV^+^ DLBCL in the GI tract [11,12,13,35,98,101]. We recently analyzed clinicopathological findings in Japanese patients with primary giDLBCL [11,12,35,101]. Among 240 gDLBCL patients, 25 (10%) harbored EBV on >80% of their tumor cells in EBER-ISH [11]. The median age was 69 years (range, 37–85 years) with EBV latency II and III in 18% and 55%, respectively. Although we found no significant difference in clinical findings between EBV^+^ and EBV-negative gDLCBL cases, the adverse impact of EBV on survival was confirmed in patients with gDLBCL treated with rituximab-containing chemotherapy (5-year OS: 58% vs. 84%; 5-year PFS: 47% vs. 77%). However, EBV^+^ gDLBCL cases with a single gastric lesion in Lugano stage I had extremely favorable outcomes, which are discussed below.

Moreover, among 62 iDLBCL patients, 10 (16%) harbored EBV on >80% of their tumor cells [12]. EBV latency II and III were each found in three patients. Interestingly, 7 (70%) cases were related to treated lymphoma (peripheral T-cell lymphoma (n = 2), classic Hodgkin lymphoma (n = 2)) or iatrogenic immunodeficiency (MTX (n = 1), IFX (n = 1), and Tac (n = 1)). Such events related to iatrogenic immunodeficiency were not found in EBV-negative giDLBCL, except for one case of EBV-negative gDLBCL with a history of extranodal NK/T-cell lymphoma, nasal type. This close association of EBV^+^ DLBCL with immunological deterioration appeared to be restricted to the intestine, but not in stomach. These EBV^+^ iDLBCL patients also presented aggressive clinical features (performance status 2–4, International Prognostic Index high-intermediate/high, and multiple intestinal lesions). The worse survival of an EBV^+^ subgroup was found with iDLBCL and gDLBCL cases.

Based on this original series, we further divided EBV^+^ giDLBCL into immunosuppressed (IS), non-immunosuppressed (non-IS) with Lugano stage I, and non-IS with Lugano stage II1/II2/IIE/IV [101]. In the non-IS with Lugano I group, the anatomical site of involvement was restricted to the stomach. Interestingly, this group showed a favorable outcome, including one unusual patient with EBV^+^ gDLBCL and a polypoid mass who had spontaneous regression 8 weeks after diagnosis without any treatment. This patient was speculated to bear an aspect of EBVMCU despite the polypoid, but not ulcerative, tumoral appearance. Unfortunately, no data were available regarding the natural long-term outcomes because most of the patients subsequently received rituximab-containing chemotherapy depending on their diagnosis of EBV^+^ gDLBCL. The issue of whether EBV^+^ giDLBCL in stage I may be classifiable as a polypoid/tumorous variant of EBVMCU in light of the favorable clinical course should be clarified in the future. When EBVMCU and EBV^+^ DLBCL are indistinguishable based on histopathology alone, special attention should be paid to their gross endoscopic appearance, especially a sharply circumscribed ulcer and/or polypoid pattern, to identify cases with a solitary gastric EBV^+^ B-cell lymphoproliferative lesions. To avoid excessive treatment, we recommended conservative management for 8 weeks in patients with favorable international prognostic index scores as a pragmatic approach. Of course, this should be validated in the future.

#### 4.4.2. Pathological Features of Primary EBV^+^ giDLBCL

In general, EBV^+^ DLBCL exhibits a broad range of morphological features. Many EBV^+^ giDLBCL cases are featured by monomorphous large B-cell proliferation and may have polymorphous infiltrate of HRS-like cells in an inflammatory background composed mainly of histiocytes and lymphocytes. Geographic necrosis and angioinvasion are common features. Most cases have a post-germinal center/activated B-cell phenotype. The majority of tumor cells express B-cell markers such as CD19, CD20, and CD79a, which are preserved with coexpression of PAX5, Oct-2, Bob.1, and MUM1. CD30 is usually positive. EBV^+^ DLBCL, NOS has been reported to exhibit prominent nuclear factor κB and JAK/STAT pathway activation in the neoplastic cells [102,103].

#### 4.4.3. PD-L1 Expression of Primary EBV^+^ giDLBCL

In our series of giDLBCL patients using PD-L1 (by clone SP142) immunohistochemistry, neoplastic cell staining was considered positive for PD-L1 (nPD-L1) when ≥5% of the lymphoid cells exhibited moderate to strong membrane staining. In addition, microenvironment immune cell staining was considered positive for PD-L1 (miPD-L1) when, among the total tissue cellularity, ≥20% comprised non-malignant cells with moderate or strong membrane or cytoplasmic PD-L1-specific staining.

In 174 giDLBCL patients in our series, we detected 2 (17%) nPD-L1^+^ cases among 12 EBV^+^ cases, and 1 (0.6%) nPD-L1^+^ case among the 162 EBV-negative cases. These figures were lower than those reported by Suzuki et al. [104], who reported 4/8 (50%) EBV^+^ extranodal DLBCL, NOS and 6/108 (6%) extranodal EBV-negative DLBCL cases positive for PD-L1 using the same PD-L1-specific antibody and cut-off value. The current hypothesis is that the divergent neoplastic PD-L1 positivity represents the vulnerability of specific anatomical sites to PD-L1-positive DLBCL [105].

**Figure 2:**
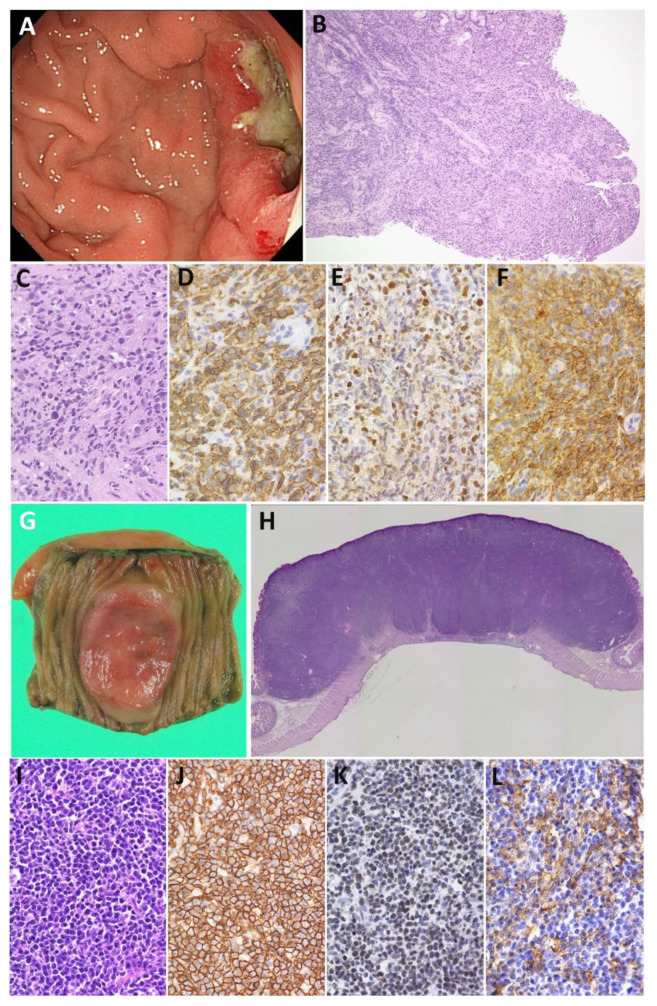
Comparison of PD-L1 expression on tumor cells and nonmalignant immune cells. (**A**–**F**) EBV^+^ gDLBCL with PD-L1 expression on tumor cells. (**A**) Endoscopic image showing an ulcerated mass in the antrum of the stomach. (**B**,**C**) Biopsy specimen revealed diffuse lymphoid proliferation of medium to large cells. (**D**) The tumor cells were positive for CD20, (**E**) EBER, and (**F**) PD-L1. (**G**–**L**) EBV^+^ iDLBCL with PD-L1 expression on microenvironment immune cells. (**G**) Surgically resected specimen has an ulcerated mass in the jejunum. (**H**) Well circumscribed tumor mass with invasion of the muscularis propria. (**I**) Diffuse proliferation of mononuclear cells. (**J**) The tumor cells were strongly and uniformly positive for CD20 and (**K**) EBER. (**L**) PD-L1 expression was detected in immune cells, but not in tumor cells. Original magnifications: ×100 (**B**) and ×200 (**C**–**F**,**I**–**L**).

EBV^+^ giDLBCL patients with nPD-L1 expression present an aggressive clinical course [35]. One EBV^+^ gDLBCL case exhibiting progressive disease after rituximab-containing chemotherapy despite Lugano stage I underwent autologous peripheral blood stem cell transplantation (Figure 2A–F). The other EBV^+^ iDLBCL case in Lugano stage II2 achieved a partial response by treatment with rituximab-containing chemotherapy but had a relapse and died of disease 10 months after diagnosis.

Moreover, our EBV^+^ giDLBCL series had high frequency of miPD-L1 expression, accounting for approximately 90% [101] (Figure 2G–L). The association of EBV^+^ DLBCL and miPD-L1 expression was also reported by Kiyasu et al. [34]. However, among patients with EBV-negative giDLBCL, miPD-L1 expression is associated with a better outcome compared to miPD-L1 negative patients. EBV-negative iDLBCL cases with high miPD-L1 expression (>40% of immune cells) especially had a favorable outcome, with a plateau in the survival curve, whereas those with PD-L1 expression on <5% of microenvironment immune cells had extremely worse survival in the current rituximab era.

Thus far in the English literature, we first documented that PD-L1 expression on non-malignant immune cells, such as macrophages and dendritic cells, contributes to better outcomes in giDLBCL patients treated with modern immunochemotherapy.

## 5. Conclusions

Evidence indicates that giEBVMCU frequently occurs in the colorectum and usually has an indolent and self-limited clinical course. EBVMCU should be considered in the differential diagnosis when patients in the setting of immunosuppression have ulcerating mucosal lesions without tumor mass and no detectable lesions in other sites on thorough imaging studies. Although EBVMCU is defined by a localized ulcer, the spectrum of EBVMCU in the GI tract may be extended to extranodal EBV^+^ LPD confined to a single anatomical site with not only solitary, but also multiple lesions or not only ulcerative, but also mass lesions in the future. We need to pay attention to the high frequency of EBV among DLBCL patients in the setting of treated lymphoma-associated or other iatrogenic immunodeficiencies. It is essential to recognize the clinicopathological spectrum between the two distinct diseases, EBVMCU and EBV^+^ DLBCL, in order to provide appropriate treatment in routine practice. In addition, we hope that treatment strategies are developed in the near future because some of the patients with giEBVMCU receive aggressive treatment despite the indolent clinical course.

EBV^+^ giDLBCL presents an aggressive clinical course, but EBV^+^ gDLBCL cases with a single lesion in Lugano stage I have extremely favorable outcomes, which may indicate that this subgroup is distinctive and possibly classifiable as EBVMCU. In contrast to giEBVMCU, EBV^+^ giDLBCL has a strong correlation with PD-L1 expression on tumor cells or immune cells. This pathological feature could aid in diagnosis and be useful for assessing either immune escape or immunodeficiency in the pathogenesis of EBV^+^ B-LPDs.

## Figures and Tables

**Table 1:** Summary of reported cases of EBVMCU of the GI tract (n = 30).

Age/Sex	Site	Endoscopic Finding	No. Lesions	Clinical Setting	Source of IS	Treatment	Outcome (Length of CR)	Length of FU (mo)	Ref
57 F	Rectum	Nonspecific erythema without ulcers	NA	IBD	(Only mesalazine)	FU	SD	6	[30]
26 M	Rectum	Large and deep ulcer	Single	IBD (CD, 11y)	AZA+IFX	RIS+Sur	CR	12	[28]
63 M	Anus	Large superficial perianal wound	Single	IBD (CD, 30y)	AZA	RIS	CR (at 6 wks)	NA	[26]
53 F	Colon, rectum	Multiple ulceration	Multiple	IBD (CD, 6y)	MTX+IFX	RIS	SD, progression to HL	18	[27]
34 M	Colon, rectum	Small and large ulcers	Multiple	IBD (UC)	6-MP	RIS+R	CR	12	[29]
78 M	Rectum	Anorectal ulcer	Single	IBD (UC)	CYA	RIS	CR	23	[25]
61 M	Esophagus	Well-circumscribed mucosal ulcer	NA	Renal transplantation	PSL+MMF	RIS	CR (at 4 wks)	16.5	[57]
29 M	Colon	Ulcerative necrotic lesion	Single	Renal transplantation	PSL+MMF+CYA	RIS+R	CR (at 5 mos)	11	[68]
27 M	Colon, rectum	Superficial lesions	Multiple	Renal transplantation	PSL+MMF+CYA	RIS+R	PD→CR *	13	[68]
70 M	Rectum	Well-circumscribed mucosal ulcer	NA	Renal transplantation	PSL+MMF	RIS+R+Velcade	CR (at 12 wks), but DOC	17	[57]
32 M	Terminal ileum	Well-circumscribed mucosal ulcer	NA	Lung transplantation	PSL+MMF+Tac	RIS+R	CR (at 4 wks), but DOC	60	[57]
69 M	Colon	Well-defined punched-out ulcers	Multiple	Melanoma (Ipi), irColitis	PSL+IFX	Sur (perforation)	CR	>60	[31]
66 M	Colon	Well-defined punched-out ulcers	Multiple	Melanoma (Ipi+Nivo), irColitis	PSL	Sur (perforation)	CR	25	[31]
70 M	Small bowel, colon, rectum	Well-defined punched-out ulcers	Multiple	Melanoma (Ipi), irColitis	PSL	Sur (perforation)	CR	>50	[31]
77 M	Colon, rectum	Well-defined punched-out ulcers	Multiple	Melanoma (Ipi), irColitis	PSL	Sur (perforation)	PD (died of perforation)	<1	[31]
75 F	Esophagus	Esophageal ulcer	Single	RA	AZA	RIS	CR	17	[25]
81 F	Jejunum	Ulcerative lesion	Single	RA	PSL+MTX	Sur (perforation)	CR	24	[67]
69 F	Colon	Colonic mass	Single	RA	MTX	NA	NA	NA	[25]
51 F	Stomach	Small shallow ulcer	Multiple	ATLL	mLSG15	FU	CR (at 4 wks), but DOC	4	[58]
35 F	Ileum, colon, rectum	NA	Multiple	ATLL	mLSG15, CHASE, M, HSCT, Tac	RIS	CR (at 12 wks)	19	[58]
38 M	Colon, rectum	Multiple ulcers and elevated lesions	Multiple	ED, cHL	ABVD	FU	NA	NA	[66]
81 F	Colon	NA	NA	AITP	PSL+AZA	Sur (perforation)	CR, but DOC	1	[65]
70 M	Rectum	Tumoral lesion	NA	HIV	HIV	FU	CR	9	[30]
64 F	Colon	Small shallow ulcer	Single	HSCT(ET, sMDS)	CYA	RIS	CR	6	[25]
61 F	Esophagus	Esophageal ulcer	Multiple	Hypogammaglobulinemia	PI	R+IVIG+B	PD	<6	[69]
83 F	Colon	Sharply circumscribed mucosal ulcer	Single	RP	PSL+MTX	Sur (diverticulitis)	CR	4	[71]
84 F	Colon	NA	Multiple		Age	Sur (diverticulitis)	CR	NA	[64]
84 F	Esophagus	NA	Single		Age	FU	CR	6	[70]
64 F	Ileocecum	Partially necrotic ulcer	Single		Age	Sur	CR, but DOC	6	[63]
81 M	Colon	Tumor with circumferential ulcer	NA		Age	Sur (obstruction)	CR	20	[62]

ABVD: doxorubicin, bleomycin, vinblastine, and dacarbazine chemotherapy; AITP: autoimmune thrombocytopenia; ATLL: adult T-cell leukemia/lymphoma; AZA: azathioprine; B: brentuximab; CD: Crohn’s disease; CHASE: cyclophosphamide, etoposide, cytarabine, and dexamethasone; cHL: classic Hodgkin lymphoma; CR: complete remission; CYA: cyclosporin-A; DOC: died of other cause; ED: epiphyseal dysplasia; ET: essential thrombocythemia; FU: follow-up; HIV: human immunodeficiency virus; HL: Hodgkin lymphoma; HSCT: hematopoietic stem cell transplantation; IBD: inflammatory bowel disease; IFX: infliximab; Ipi: Ipilimumab (anti-CTLA-4); irColitis: immune-related colitis; IS: immune suppression; IVIG: intravenous immunoglobulin; M: mogalizumab; mLSG15: modified LSG 15 chemotherapy; MMF: mycophenolate mofetil; 6-MP: 6-mercaptopurine; MTX: methotrexate; NA: not available; Nivo: nivolumab (anti-PD-1); PD: progressive disease; PI: primary immunodeficiency; PSL: prednisolone; R: rituximab; RA: rheumatoid arthritis; RIS: reduction of IS; RP: rheumatic polymyalgias; S: sigmoid colon; SD: stable disease; sMDS: secondary myelodysplasia; Sur: surgery; Tac: tacrolimus; UC: ulcerative colitis. * 10 months after IS tapering and R therapy, CYA and MMF were withdrawn because of digestive bleeding, which resulted in CR after 3 months.

**Table 2:** Clinicopathological features of 30 cases with EBVMCU of the GI tract.

Male	16	(55%)	**Source of IS**		
Median age, years (range)	65	(26–84)	AZA or 6-MP	3	(10%)
**Site**			AZA+IFX	1	(3%)
Esophagus	4	(13%)	CYA	2	(7%)
Stomach	1	(3%)	MTX	2	(7%)
Small intestine	2	(7%)	MTX+IFX	1	(3%)
Ileocecum	1	(3%)	PSL	3	(10%)
Colon	9	(30%)	PSL+AZA, IFX, MMF, or MTX	5	(17%)
Rectum	5	(17%)	PSL+MMF+CYA or Tac	3	(10%)
Colon, rectum	6	(20%)	CTx	3	(10%)
Small intestine, colon, rectum	1	(3%)	Age	4	(13%)
Anus	1	(3%)	Others	2	(7%)
**The number of lesions**			None	1	(3%)
Single lesion	11	(55%)	**Treatment**		
Multiple lesions	9	(45%)	RIS	8	(27%)
NA	10		RIS+R	4	(13%)
**Clinical setting**			RIS+CTx	2	(7%)
IBD	6	(20%)	Surgery	10	(33%)
Organ transplant	5	(17%)	Follow up	5	(17%)
irColitis	4	(13%)	NA	1	(3%)
RA	3	(10%)	**Outcome**		
Treated-lymphoma	3	(10%)	CR	22	(73%)
Old age	4	(13%)	SD	2	(7%)
Others	5	(17%)	PD	4	(13%)
			NA	2	(7%)

Data are given as n (%) unless otherwise noted. AZA: azathioprine; CR: complete remission; CYA: cyclosporin-A; IBD: inflammatory bowel disease; IFX: infliximab; irColitis: immune-related colitis; IS: immune suppression; 6-MP: 6-mercaptopurine; MTX: methotrexate; NA: not available; PD: progressive disease; PSL: prednisolone; R: rituximab; RA: rheumatoid arthritis; RIS: reduction of IS; SD: stable disease; Tac: tacrolimus.

## References

[1] Dojcinov S.D., Fend F., Quintanilla-Martinez L. (2018). EBV-Positive Lymphoproliferations of B-T-and NK-Cell Derivation in Non-Immunocompromised Hosts. Pathogens.

[2] Luzuriaga K., Sullivan J.L. (2010). Infectious Mononucleosis. N. Engl. J. Med..

[3] Kimura H., Cohen J.I. (2017). Chronic Active Epstein-Barr Virus Disease. Front. Immunol..

[4] Tian S.F., Westbrook L.M., Xiao S.Y., Zhang Y.L., Huang Y., Wang H.L.L. (2019). The Morphologic Features of Primary Epstein-Barr Virus Infection in the Gastrointestinal Tract an Approach to Correct Diagnosis. Am. J. Surg. Pathol..

[5] Xu W.J., Jiang X.Y., Chen J.J., Mao Q.Q., Zhao X.G., Sun X., Zhong L., Rong L. (2020). Chronic active Epstein-Barr virus infection involving gastrointestinal tract mimicking inflammatory bowel disease. BMC Gastroenterol..

[6] Oyama T., Ichimura K., Suzuki R., Suzumiya J., Ohshima K., Yatabe Y., Yokoi T., Kojima M., Kamiya Y., Taji H. (2003). Senile EBV plus B-cell Lymphoproliferative disorders—A clinicopathologic study of 22 patients. Am. J. Surg. Pathol..

[7] Oyama T., Yamamoto K., Asano N., Oshiro A., Suzuki R., Kagami Y., Morishima Y., Takeuchi K., Izumo T., Mori S. (2007). Age-related EBV-Associated B-Cell Lymphoproliferative disorders constitute a distinct clinicopathologic group: A study of 96 patients. Clin. Cancer Res..

[8] Park S., Lee J., Ko Y.H., Han A., Jun H.J., Lee S.C., Hwang I.G., Park Y.H., Ahn J.S., Jung C.W. (2007). The impact of Epstein-Barr virus status on clinical outcome in diffuse large B-cell lymphoma. Blood.

[9] Morales D., Beltran B., De Mendoza F.H., Riva L., Yabar A., Quinones P., Butera J.N., Castillo J. (2010). Epstein-Barr virus as a prognostic factor in de novo nodal diffuse large B-cell lymphoma. Leuk. Lymphoma.

[10] Nicolae A., Pittaluga S., Abdullah S., Steinberg S.M., Pham T.A., Davies-Hill T., Xi L.Q., Raffeld M., Jaffe E.S. (2015). EBV-positive large B-cell lymphomas in young patients: A nodal lymphoma with evidence for a tolerogenic immune environment. Blood.

[11] Ishikawa E., Tanaka T., Shimada K., Kohno K., Satou A., EladI A.E., Sakakibara A., Furukawa K., Funasaka K., Miyahara R. (2018). A prognostic model, including the EBV status of tumor cells, for primary gastric diffuse large B-cell lymphoma in the rituximab era. Cancer Med..

[12] Ishikawa E., Kato S., Shimada K., Tanaka T., Suzuki Y., Satou A., Kohno K., Sakakibara A., Yamamura T., Nakamura M. (2018). Clinicopathological analysis of primary intestinal diffuse large B-cell lymphoma: Prognostic evaluation of CD5, PD-L1, and Epstein-Barr virus on tumor cells. Cancer Med..

[13] Maeshima A.M., Taniguchi H., Ito Y., Hatta S., Suzuki T., Yuda S., Makita S., Fukuhara S., Munakata W., Suzuki T. (2019). Clinicopathological characteristics of diffuse large B-cell lymphoma involving small and large intestines: An analysis of 126 patients. Int. J. Hematol..

[14] Ahn J.S., Yang D.H., Choi Y.D., Jung S.H., Yhim H.Y., Kwak J.Y., Park H.S., Shin M.G., Kim Y.K., Kim H.J. (2013). Clinical outcome of elderly patients with Epstein-Barr virus positive diffuse large B-cell lymphoma treated with a combination of rituximab and CHOP chemotherapy. Am. J. Hematol..

[15] Sato A., Nakamura N., Kojima M., Ohmachi K., Carreras J., Kikuti Y.Y., Numata H., Ohgiya D., Tazume K., Amaki J. (2014). Clinical outcome of Epstein-Barr virus-positive diffuse large B-cell lymphoma of the elderly in the rituximab era. Cancer Sci..

[16] Hong J.Y., Yoon D.H., Suh C., Huh J., Do I.G., Sohn I., Jo J., Jung S.H., Hong M.E., Yoon H. (2015). EBV-positive diffuse large B-cell lymphoma in young adults: Is this a distinct disease entity?. Ann. Oncol..

[17] Ok C.Y., Ye Q., Li L., Manyam G.C., Deng L.J., Goswami R.R., Wang X.X., Montes-Moreno S., Visco C., Tzankov A. (2015). Age cutoff for Epstein-Barr virus-positive diffuse large B-cell lymphoma—is it necessary?. Oncotarget.

[18] Dojcinov S.D., Venkataraman G., Raffeld M., Pittaluga S., Jaffe E.S. (2010). EBV Positive Mucocutaneous Ulcer-A Study of 26 Cases Associated with Various Sources of Immunosuppression. Am. J. Surg. Pathol..

[19] Matnani R., Peker D. (2014). Azathioprine induced Epstein Barr virus-positive mucocutaneous ulcer arising in perianal fistula and abscess associated with Crohn’s disease. J. Crohns Colitis.

[20] Moran N.R., Webster B., Lee K.M., Trotman J., Kwan Y.L., Napoli J., Leong R.W. (2015). Epstein Barr virus-positive mucocutaneous ulcer of the colon associated Hodgkin lymphoma in Crohn’s disease. World J. Gastroenterol..

[21] Juan A., Lobaton T., Tapia G., Manosa M., Cabre E., Domenech E. (2017). Epstein-Barr virus-positive mucocutaneous ulcer in Crohn’s disease. A condition to consider in immunosuppressed IBD patients. Dig. Liver Dis..

[22] Goetgebuer R.L., van der Woude C.J., de Ridder L., Doukas M., de Vries A.C. (2019). Clinical and endoscopic complications of Epstein-Barr virus in inflammatory bowel disease: An illustrative case series. Int. J. Colorectal Dis..

[23] Prieto-Torres L., Erana I., Gil-Redondo R., de la Riva I.G., Manso R., Pajares R., Cordoba R., Machan S., Ara M., Requena L. (2019). The Spectrum of EBV-Positive Mucocutaneous Ulcer A Study of 9 Cases. Am. J. Surg. Pathol..

[24] Pugh M.R., Leopold G.D., Morgan M., Christian A.D., Hewett R., Durai D., Wagstaff J., Harris D., Dojcinov S.D. (2020). Epstein-Barr Virus-Positive Mucocutaneous Ulcers Complicate Colitis Caused by Immune Checkpoint Regulator Therapy and Associate with Colon Perforation. Clin. Gastroenterol. Hepatol..

[25] Narimatsu H., Ota Y., Kami M., Takeuchi K., Suzuki R., Matsuo K., Matsumura T., Yuji K., Kishi Y., Hamaki T. (2007). Clinicopathological features of pyothorax-associated lymphoma; a retrospective survey involving 98 patients. Ann. Oncol..

[26] Song J.Y., Pittaluga S., Dunleavy K., Grant N., White T., Jiang L.Y., Davies-Hill T., Raffeld M., Wilson W.H., Jaffe E.S. (2015). Lymphomatoid Granulomatosis-A Single Institute Experience Pathologic Findings and Clinical Correlations. Am. J. Surg. Pathol..

[27] Beaty M.W., Toro J., Sorbara L., Stern J.B., Pittaluga S., Raffeld M., Wilson W.H., Jaffe E.S. (2001). Cutaneous lymphomatoid granulomatosis—Correlation of clinical and biologic features. Am. J. Surg. Pathol..

[28] Dong H.D., Strome S.E., Salomao D.R., Tamura H., Hirano F., Flies D.B., Roche P.C., Lu J., Zhu G.F., Tamada K. (2002). Tumor-associated B7-H1 promotes T-cell apoptosis: A potential mechanism of immune evasion. Nat. Med..

[29] Chen B.J., Chapuy B., Jing O.Y., Sun H.H., Roemer M.G.M., Xu M.L., Yu H.B., Fletcher C.D.M., Freeman G.J., Shipp M.A. (2013). PD-L1 Expression Is Characteristic of a Subset of Aggressive B-cell Lymphomas and Virus-Associated Malignancies. Clin. Cancer Res..

[30] Brahmer J.R., Tykodi S.S., Chow L.Q.M., Hwu W.J., Topalian S.L., Hwu P., Drake C.G., Camacho L.H., Kauh J., Odunsi K. (2012). Safety and Activity of Anti-PD-L1 Antibody in Patients with Advanced Cancer. N. Engl. J. Med..

[31] Balar A.V., Weber J.S. (2017). PD-1 and PD-L1 antibodies in cancer: Current status and future directions. Cancer Immunol. Immunother..

[32] Lesokhin A.M., Ansell S.M., Armand P., Scott E.C., Halwani A., Gutierrez M., Millenson M.M., Cohen A.D., Schuster S.J., Lebovic D. (2016). Nivolumab in Patients with Relapsed or Refractory Hematologic Malignancy: Preliminary Results of a Phase Ib Study. J. Clin. Oncol..

[33] Sakakibara A., Kohno K., Ishikawa E., Suzuki Y., Shimada S., Eladl A.E., Elsayed A.A., Daroontum T., Satou A., Takahara T. (2020). Age-related EBV-associated B-cell lymphoproliferative disorders and other EBV plus lymphoproliferative diseases: New insights into immune escape and immunodeficiency through staining with anti-PD-L1 antibody clone SP142. Pathol. Int..

[34] Kiyasu J., Miyoshi H., Hirata A., Arakawa F., Ichikawa A., Niino D., Sugita Y., Yufu Y., Choi I., Abe Y. (2015). Expression of programmed cell death ligand 1 is associated with poor overall survival in patients with diffuse large B-cell lymphoma. Blood.

[35] Ishikawa E., Nakamura M., Shimada K., Tanaka T., Satou A., Kohno K., Sakakibara A., Furukawa K., Yamamura T., Miyahara R. (2020). Prognostic impact of PD-L1 expression in primary gastric and intestinal diffuse large B-cell lymphoma. J. Gastroenterol..

[36] Thorley-Lawson D.A., Gross A. (2004). Mechanisms of disease—Persistence of the Epstein-Barr virus and the origins of associated lymphomas. N. Engl. J. Med..

[37] Taylor G.S., Long H.M., Brooks J.M., Rickinson A.B., Hislop A.D. (2015). The immunology of Epstein-Barr Virus Induced Disease. Annu. Rev. Immunol..

[38] Young L.S., Rickinson A.B. (2004). Epstein-Barr virus: 40 years on. Nat. Rev. Cancer.

[39] Young L.S., Yap L.F., Murray P.G. (2016). Epstein-Barr virus: More than 50 years old and still providing surprises. Nat. Rev. Cancer.

[40] Satou A., Asano N., Nakazawa A., Osumi T., Tsurusawa M., Ishiguro A., Elsayed A.A., Nakamura N., Ohshima K., Kinoshita T. (2015). Epstein-Barr Virus (EBV)-positive Sporadic Burkitt Lymphoma an Age-related Lymphoproliferative Disorder?. Am. J. Surg. Pathol..

[41] Ryan J.L., Shen Y.J., Morgan D.R., Thorne L.B., Kenney S.C., Dominguez R.L., Gulley M.L. (2012). Epstein-Barr Virus Infection Is Common in Inflamed Gastrointestinal Mucosa. Dig. Dis. Sci..

[42] Yanai H., Shimizu N., Nagasaki S., Mitani N., Okita K. (1999). Epstein-Barr virus infection of the colon with inflammatory bowel disease. Am. J. Gastroenterol..

[43] Shukla S.K., Prasad K.N., Tripathi A., Singh A., Saxena A., Chand Ghoshal U., Krishnani N., Husain N. (2011). Epstein-Barr virus DNA load and its association with Helicobacter pylori infection in gastroduodenal diseases. Braz. J. Infect. Dis..

[44] Yanai H., Murakami T., Yoshiyama H., Takeuchi H., Nishikawa J., Nakamura H., Okita K., Miura O., Shimizu N., Takada K. (1999). Epstein-Barr virus-associated gastric carcinoma and atrophic gastritis. J. Clin. Gastroenterol..

[45] Bass A.J., Thorsson V., Shmulevich I., Reynolds S.M., Miller M., Bernard B., Hinoue T., Laird P.W., Curtis C., Shen H. (2014). Comprehensive molecular characterization of gastric adenocarcinoma. Nature.

[46] Saito R., Abe H., Kunita A., Yamashita H., Seto Y., Fukayama M. (2017). Overexpression and gene amplification of PD-L1 in cancer cells and PD-L1(+) immune cells in Epstein-Barr virus-associated gastric cancer: The prognostic implications. Mod. Pathol..

[47] Fang W.L., Chen M.H., Huang K.H., Lin C.H., Chao Y., Lo S.S., Li A.F.Y., Wu C.W., Shyr Y.M. (2020). The Clinicopathological Features and Genetic Alterations in Epstein-Barr Virus-Associated Gastric Cancer Patients after Curative Surgery. Cancers.

[48] Kim S.T., Cristescu R., Bass A.J., Kim K.M., Odegaard J.I., Kim K., Liu X.Q., Sher X.W., Jung H., Lee M. (2018). Comprehensive molecular characterization of clinical responses to PD-1 inhibition in metastatic gastric cancer. Nat. Med..

[49] Kono K., Nakajima S., Mimura K. (2020). Current status of immune checkpoint inhibitors for gastric cancer. Gastric Cancer.

[50] Sankaran-Walters S., Ransibrahmanakul K., Grishina I., Hung J., Martinez E., Prindiville T., Dandekar S. (2011). Epstein-Barr virus replication linked to B cell proliferation in inflamed areas of colonic mucosa of patients with inflammatory bowel disease. J. Clin. Virol..

[51] Lopes S., Andrade P., Conde S., Liberal R., Dias C.C., Fernandes S., Pinheiro J., Simoes J.S., Carneiro F., Magro F. (2017). Looking into Enteric Virome in Patients with IBD: Defining Guilty or Innocence?. Inflamm. Bowel Dis..

[52] Dayharsh G.A., Loftus E.V., Sandborn W.J., Tremaine W.J., Zinsmeister A.R., Witzig T.E., Macon W.R., Burgart L.J. (2002). Epstein-Barr virus-positive lymphoma in patients with inflammatory bowel disease treated with azathioprine or 6-mercaptopurine. Gastroenterology.

[53] Beaugerie L., Brousse N., Bouvier A.M., Colombel J.F., Lemann M., Cosnes J., Hebuterne X., Cortot A., Bouhnik Y., Gendre J.P. (2009). Lymphoproliferative disorders in patients receiving thiopurines for inflammatory bowel disease: A prospective observational cohort study. Lancet.

[54] Lemaitre M., Kirchgesner J., Rudnichi A., Carrat F., Zureik M., Carbonnel F., Dray-Spira R. (2017). Association Between Use of Thiopurines or Tumor Necrosis Factor Antagonists Alone or in Combination and Risk of Lymphoma in Patients with Inflammatory Bowel Disease. JAMA J. Am. Med. Assoc..

[55] Rezk S.A., Weiss L.M. (2007). Epstein-Barr virus-associated lymphoproliferative disorders. Hum. Pathol..

[56] Ghia P., Prato G., Stella S., Scielzo C., Geuna M., Caligaris-Cappio F. (2007). Age-dependent accumulation of monoclonal CD4(+)CD8(+)double positive T lymphocytes in the peripheral blood of the elderly. Br. J. Haematol..

[57] Ikeda T., Gion Y., Sakamoto M., Tachibana T., Nishikori A., Nishimura M.F., Yoshino T., Sato Y. (2020). Clinicopathological analysis of 34 Japanese patients with EBV-positive mucocutaneous ulcer. Mod. Pathol..

[58] Hart M., Thakral B., Yohe S., Balfour H.H., Singh C., Spears M., McKenna R.W. (2014). EBV-positive Mucocutaneous Ulcer in Organ Transplant Recipients a Localized Indolent Posttransplant Lymphoproliferative Disorder. Am. J. Surg. Pathol..

[59] Satou A., Banno S., Hanamura I., Takahashi E., Takahara T., Nobata H., Katsuno T., Takami A., Ito Y., Ueda R. (2019). EBV-positive mucocutaneous ulcer arising in rheumatoid arthritis patients treated with methotrexate: Single center series of nine cases. Pathol. Int..

[60] Daroontum T., Kohno K., Eladl A.E., Satou A., Sakakibara A., Matsukage S., Yakushiji N., Ya-In C., Nakamura S., Asano N. (2018). Comparison of Epstein-Barr virus-positive mucocutaneous ulcer associated with treated lymphoma or methotrexate in Japan. Histopathology.

[61] Daroontum T., Kohno K., Inaguma Y., Okamoto A., Okamoto M., Kimura Y., Nagahama M., Sakakibara A., Satou A., Nakamura S. (2019). Epstein-Barr virus (EBV)-positive diffuse large B-cell lymphoma arising in patient with a history of EBV-positive mucocutaneous ulcer and EBV-positive nodal polymorphous B-lymphoproliferative disorder. Pathol. Int..

[62] Sinit R.B., Horan K.L., Dorer R.K., Aboulafia D.M. (2019). Epstein-Barr Virus-Positive Mucocutaneous Ulcer: Case Report and Review of the First 100 Published Cases. Clin. Lymphoma Myeloma Leuk..

[63] Morita N., Okuse C., Suetani K., Nakano H., Hiraishi T., Ishigooka S., Mori S., Shimamura T., Asakura T., Koike J. (2020). A rare case of Epstein-Barr virus-positive mucocutaneous ulcer that developed into an intestinal obstruction: A case report. BMC Gastroenterol..

[64] Osman M., Al Salihi M., Abu Sitta E., Al Hadidi S. (2017). A rare case of Epstein-Barr virus mucocutaneous ulcer of the colon. BMJ Case Rep..

[65] Zanelli M., Zizzo M., Foroni M., De Marco L., Martino G., Ascani S. (2019). EBV-positive mucocutaneous ulcer within colonic diverticulitis mimicking diffuse large B cell lymphoma. Ann. Hematol..

[66] Di Napoli A., Giubettini M., Duranti E., Ferrari A., Guglielmi C., Uccini S., Ruco L. (2011). Iatrogenic EBV-positive lymphoproliferative disorder with features of EBV plus mucocutaneous ulcer: Evidence for concomitant TCR gamma/IGH rearrangements in the Hodgkin-like neoplastic cells. Virchows Archiv.

[67] Karube K., Takatori M., Kohno K., Tomoyose T., Ohshiro K., Nakazato I. (2020). Co-occurrence of EBV-positive classic Hodgkin lymphoma and B-cell lymphomas of different clonal origins: A case report and literature review. Pathol. Int..

[68] Nomura M., Sumiya R., Ono H., Nagai T., Kumazawa K., Shimizu A., Endo D., Aoyanagi N. (2021). Cessation of methotrexate and a small intestinal resection provide a good clinical course for a patient with a jejunum perforation induced by a methotrexate-associated lymphoproliferative disorder: A case report. World J. Surg. Oncol..

[69] Isnard P., Bruneau J., Sberro-Soussan R., Wendum D., Legendre C., Molina T., Chatenoud L., Hermine O., Rossignol J. (2021). Dissociation of humoral and cellular immune responses in kidney transplant recipients with EBV mucocutaneous ulcer. Transpl. Infect. Dis..

[70] Kleinman S., Jhaveri D., Caimi P., Cameron R., Lemonovich T., Meyerson H., Hostoffer R., Tcheurekdjian H. (2014). A rare presentation of EBV+ mucocutaneous ulcer that led to a diagnosis of hypogammaglobulinemia. J. Allergy Clin. Immunol. Pract..

[71] Kim C.H., Chapman J.R., Vega F. (2019). A case of EBV-associated blastic lymphoplasmacytic proliferation in an oesophageal ulcer with a self-limiting course: Overlapping lesion between EBV mucocutaneous ulcer and polymorphic lymphoplasmacytic disorder. Histopathology.

[72] Zanelli M., Mengoli M.C., Valli R., Froio E., Bisagni A., Zizzo M., De Marco L., Ascani S. (2019). Primary classic Hodgkin lymphoma of the ileum and Epstein-Barr virus mucocutaneous ulcer of the colon: Two entities compared. Virchows Archiv.

[73] Ma S.D., Xu X.Q., Jones R., Delecluse H.J., Zumwalde N.A., Sharma A., Gumperz J.E., Kenney S.C. (2016). PD-1/CTLA-4 Blockade Inhibits Epstein-Barr Virus-Induced Lymphoma Growth in a Cord Blood Humanized-Mouse Model. PLoS Pathog..

[74] Ock C.Y., Hwang J.E., Keam B., Kim S.B., Shim J.J., Jang H.J., Park S., Sohn B.H., Cha M., Ajani J.A. (2017). Genomic landscape associated with potential response to anti-CTLA-4 treatment in cancers. Nat. Commun..

[75] Oh D.Y., Cham J., Zhang L., Fong G., Kwek S.S., Klinger M., Faham M., Fong L. (2017). Immune Toxicities Elicted by CTLA-4 Blockade in Cancer Patients Are Associated with Early Diversification of the T-cell Repertoire. Cancer Res..

[76] Hoeller S., Tzankov A., Pileri S.A., Went P., Dirnhofer S. (2010). Epstein-Barr virus positive diffuse large B-cell lymphoma in elderly patients is rare in Western populations. Hum. Pathol..

[77] Dojcinov S.D., Venkataraman G., Pittaluga S., Wlodarska I., Schrager J.A., Raffeld M., Hills R.K., Jaffe E.S. (2011). Age-related EBV-associated lymphoproliferative disorders in the Western population: A spectrum of reactive lymphoid hyperplasia and lymphoma. Blood.

[78] Asano N., Yamamoto K., Tamaru J.I., Oyama T., Ishida F., Ohshima K., Yoshino T., Nakamura N., Mori S., Yoshie O. (2009). Age-related Epstein-Barr virus (EBV)-associated B-cell lymphoproliferative disorders: Comparison with EBV-positive classic Hodgkin lymphoma in elderly patients. Blood.

[79] Olsson J., Wikby A., Johansson B., Lofgren S., Nilsson B.O., Ferguson F.G. (2000). Age-related change in peripheral blood T-lymphocyte subpopulations and cytomegalovirus infection in the very old: The Swedish longitudinal OCTO immune study. Mech. Ageing Dev..

[80] Vescovini R., Telera A., Fagnoni F.F., Biasini C., Medici M.C., Valcavi P., di Pede P., Lucchini G., Zanlari L., Passeri G. (2004). Different contribution of EBV and CMV infections in very long-term carriers to age-related alterations of CD8(+) T cells. Exp. Gerontol..

[81] Cardenas D., Velez G., Orfao A., Herrera M.V., Solano J., Olaya M., Uribe A.M., Saavedra C., Duarte M., Rodriguez M. (2015). Epstein-Barr virus-specific CD8(+) T lymphocytes from diffuse large B cell lymphoma patients are functionally impaired. Clin. Exp. Immunol..

[82] Habermann T.M., Weller E.A., Morrison V.A., Gascoyne R.D., Cassileth P.A., Cohn J.B., Dakhil S.R., Woda B., Fisher R.I., Peterson B.A. (2006). Rituximab-CHOP versus CHOP alone or with maintenance rituximab in older patients with diffuse large B-cell lymphoma. J. Clin. Oncol..

[83] Pfreundschuh M., Kuhnt E., Trumper L., Osterborg A., Trneny M., Shepherd L., Gill D.S., Walewski J., Pettengell R., Jaeger U. (2011). CHOP-like chemotherapy with or without rituximab in young patients with good-prognosis diffuse large-B-cell lymphoma: 6-year results of an open-label randomised study of the MabThera International Trial (MInT) Group. Lancet Oncol..

[84] Witte H.M., Merz H., Biersack H., Bernard V., Riecke A., Gebauer J., Lehnert H., von Bubnoff N., Feller A.C., Gebauer N. (2020). Impact of treatment variability and clinicopathological characteristics on survival in patients with Epstein-Barr-Virus positive diffuse large B cell lymphoma. Br. J. Haematol..

[85] Georgiou K., Chen L.Y., Berglund M., Ren W.C., de Miranda N., Lisboa S., Fangazio M., Zhu S.D., Hou Y., Wu K. (2016). Genetic basis of PD-L1 overexpression in diffuse large B-cell lymphomas. Blood.

[86] McCord R., Bolen C.R., Koeppen H., Kadel E.E., Oestergaard M.Z., Nielsen T., Sehn L.H., Venstrom J.M. (2019). PD-L1 and tumor-associated macrophages in de novo DLBCL. Blood Adv..

[87] Hu L.Y., Xu X.L., Rao H.L., Chen J., Lai R.C., Huang H.Q., Jiang W.Q., Lin T.Y., Xia Z.J., Cai Q.Q. (2017). Expression and clinical value of programmed cell death-ligand 1 (PD-L1) in diffuse large B cell lymphoma: A retrospective study. Chin. J. Cancer.

[88] Kataoka K., Shiraishi Y., Takeda Y., Sakata S., Matsumoto M., Nagano S., Maeda T., Nagata Y., Kitanaka A., Mizuno S. (2016). Aberrant PD-L1 expression through 3 ‘-UTR disruption in multiple cancers. Nature.

[89] Kwon D., Kim S., Kim P.J., Go H., Nam S.J., Paik J.H., Kim Y.A., Kim T.M., Heo D.S., Kim C.W. (2016). Clinicopathological analysis of programmed cell death 1 and programmed cell death ligand 1 expression in the tumour microenvironments of diffuse large B cell lymphomas. Histopathology.

[90] Green M.R., Rodig S., Juszczynski P., Ouyang J., Sinha P., O’Donnell E., Neuberg D., Shipp M.A. (2012). Constitutive AP-1 Activity and EBV Infection Induce PD-L1 in Hodgkin Lymphomas and Posttransplant Lymphoproliferative Disorders: Implications for Targeted Therapy. Clin. Cancer Res..

[91] Takahara T., Satou A., Ishikawa E., Kohno K., Kato S., Suzuki Y., Takahashi E., Ohashi A., Asano N., Tsuzuki T. (2021). Clinicopathological analysis of neoplastic PD-L1-positive EBV(+)diffuse large B cell lymphoma, not otherwise specified, in a Japanese cohort. Virchows Archiv.

[92] Nakamura S., Matsumoto T., Iida M., Yao T., Tsuneyoshi M. (2003). Primary gastrointestinal lymphoma in Japan—A clinicopathologic analysis of 455 patients with special reference to its time trends. Cancer.

[93] Papaxoinis G., Papageorgiou S., Rontogianni D., Kaloutsi V., Fountzilas G., Pavlidis N., Dimopoulos M., Tsatalas C., Xiros N., Economopoulos T. (2006). Primary gastrointestinal non-Hodgkin’s lymphoma: A clinicopathologic study of 128 cases in Greece. A Hellenic Cooperative Oncology Group study (HeCOG). Leuk. Lymphoma.

[94] Ding W.S., Zhao S., Wang J.C., Yang Q.P., Sun H., Yan J.Q., Gao L.M., Yao W.Q., Zhang W.Y., Liu W.P. (2016). Gastrointestinal Lymphoma in Southwest China: Subtype Distribution of 1,010 Cases Using the WHO (2008) Classification in a Single Institution. Acta Haematol..

[95] Chuang S.S., Ye H., Yang S.F., Huang W.T., Chen H.K., Hsieh P.P., Hwang W.S., Chang K.Y., Lu C.L., Du M.Q. (2008). Perforation predicts poor prognosis in patients with primary intestinal diffuse large B-cell lymphoma. Histopathology.

[96] Tanaka T., Shimada K., Yamamoto K., Hirooka Y., Niwa Y., Sugiura I., Kitamura K., Kosugi H., Kinoshita T., Goto H. (2012). Retrospective analysis of primary gastric diffuse large B cell lymphoma in the rituximab era: A multicenter study of 95 patients in Japan. Ann. Hematol..

[97] Kuo S.H., Yeh K.H., Chen L.T., Lin C.W., Hsu P.N., Hsu C., Wu M.S., Tzeng Y.S., Tsai H.J., Wang H.P. (2014). Helicobacter pylori-related diffuse large B-cell lymphoma of the stomach: A distinct entity with lower aggressiveness and higher chemosensitivity. Blood Cancer J..

[98] Yoshino T., Nakamura S., Matsuno Y., Ochiai A., Yokoi T., Kitadai Y., Suzumiya J., Tobinai K., Kobayashi Y., Oda I. (2006). Epstein-Barr virus involvement is a predictive factor for the resistance to chemoradiotherapy of gastric diffuse large B-cell lymphoma. Cancer Sci..

[99] Nakamura S., Ye H., Bacon C.M., Goatly A., Liu H.X., Kerr L., Banham A.H., Streubel B., Yao T., Tsuneyoshi M. (2008). Translocations involving the immunoglobulin heavy chain gene locus predict better survival in gastric diffuse large B-cell lymphoma. Clin. Cancer Res..

[100] Kim S.J., Kang H.J., Kim J.S., Oh S.Y., Choi C.W., Lee S.I., Won J.H., Kim M.K., Kwon J.H., Mun Y.C. (2011). Comparison of treatment strategies for patients with intestinal diffuse large B-cell lymphoma: Surgical resection followed by chemotherapy versus chemotherapy alone. Blood.

[101] Miyagi S., Ishikawa E., Nakamura M., Shimada K., Yamamura T., Furukawa K., Tanaka T., Mabuchi S., Tsuyuki Y., Kohno K. (2020). Reappraisal of Primary Epstein-Barr Virus (EBV)-positive Diffuse Large B-Cell Lymphoma of the Gastrointestinal Tract Comparative Analysis Among Immunosuppressed and Nonimmunosuppressed Stage I and II–IV Patients. Am. J. Surg. Pathol..

[102] Montes-Moreno S., Odqvist L., Diaz-Perez J.A., Lopez A.B., de Villambrosia S.G., Mazorra F., Castillo M.E., Lopez M., Pajares R., Garcia J.F. (2012). EBV-positive diffuse large B-cell lymphoma of the elderly is an aggressive post-germinal center B-cell neoplasm characterized by prominent nuclear factor-kB activation. Mod. Pathol..

[103] Ok C.Y., Li L., Xu-Monette Z.Y., Visco C., Tzankov A., Manyam G.C., Montes-Moreno S., Dybaer K., Chiu A., Orazi A. (2014). Prevalence and Clinical Implications of Epstein-Barr Virus Infection in De Novo Diffuse Large B-Cell Lymphoma in Western Countries. Clin. Cancer Res..

[104] Suzuki Y., Sakakibara A., Shimada K., Shimada S., Ishikawa E., Nakamura S., Kato S., Takahara T., Asano N., Satou A. (2019). Immune evasion-related extranodal large B-cell lymphoma: A report of six patients with neoplastic PD-L1-positive extranodal diffuse large B-cell lymphoma. Pathol. Int..

[105] Tsuyuki Y., Ishikawa E., Kohno K., Shimada K., Ohka F., Suzuki Y., Mabuchi S., Satou A., Takahara T., Kato S. (2021). Expression of programmed cell death ligand-1 by immune cells in the microenvironment is a favorable prognostic factor for primary diffuse large B-cell lymphoma of the central nervous system. Neuropathology.

